# Hematological ratios and cytokine profiles in heterozygous beta-thalassemia

**DOI:** 10.1016/j.htct.2025.103845

**Published:** 2025-05-13

**Authors:** Ana Carolina Marques Ciceri, Laura Eduarda de Oliveira, Ana Luísa Richter, José Antonio Mainardi de Carvalho, Maylla Rodrigues Lucena, Guilherme Wataru Gomes, Maria Stella Figueiredo, Magnun Nueldo Nunes dos Santos, Vera Lúcia Nascimento Blaia-D'Avila, Rodolfo Delfini Cançado, Elvira Maria Guerra-Shinohara, Clóvis Paniz

**Affiliations:** a Universidade Federal de Santa Maria, Departamento de Análises Clínicas e Toxicológicas, Laboratório de Pesquisas em Análises Clínicas Aplicadas - LAPACA, Santa Maria, RS, Brazil; b Universidade Ceuma - Campus Imperatriz, Imperatriz, MA, Brazil; c Universidade Federal de São Paulo, Disciplina de Hematologia e Hemoterapia, São Paulo, SP, Brazil; d Universidade Federal Fluminense, Departamento de Patologia, Niterói, RJ, Brazil; e Universidade Estadual de Campinas, Faculdade de Ciências Médicas, Departamento de Patologia Clínica, São Paulo, SP, Brazil; f Pontifícia Universidade Católica de São Paulo, Faculdade de Medicina e Ciências da Saúde, Divisão de Hematologia, Sorocaba, SP, Brazil; g Faculdade de Ciências Médicas da Santa Casa de São Paulo, Departmento de Hematologia e Oncologia, São Paulo, SP, Brazil

**Keywords:** Β-thalassemia, Heterozygotic thalassemia, Hematologic ratios, Inflammation, Hemolytic anemia

## Abstract

**Introduction:**

β-Thalassemia is defined by a reduced or complete absence of β-globin chain synthesis in hemoglobin, leading to hemolytic anemia. Heterozygous β-thalassemia, also known as β-thalassemia trait (hBTh), the mildest form of this anemia, typically does not cause symptoms in carriers. However, it may lead to changes in the immune system, including an increase in total leukocyte, neutrophil, and lymphocyte counts.

**Objective:**

This study aimed to evaluate various immune and inflammation markers, including neutrophil/lymphocyte, derived neutrophil/lymphocyte, lymphocyte/monocyte, platelet/lymphocyte, neutrophil/platelet ratios, systemic immune-inflammation index, systemic inflammation response index, neutrophil/natural killer cell ratio (NNKR), and inflammatory cytokines in β-thalassemia trait carriers.

**Method:**

A retrospective observational study was conducted, including 50 β-thalassemia trait individuals and 100 healthy controls.

**Results:**

Leukocyte, neutrophil and reticulocyte counts, and interleukin 6 levels were higher in carriers compared to controls. Notably, the β-thalassemia trait group had increased neutrophil/platelet, neutrophil/lymphocyte and derived neutrophil/lymphocyte ratios, and the systemic immune-inflammation and systemic inflammation response indexes were higher compared to the controls.

**Conclusions:**

β-thalassemia trait shows a more pronounced inflammatory profile as indicated by hematological ratios. These ratios, therefore are potentially cost-effective and easily applicable markers for monitoring patients with the β-thalassemia trait.

## Introduction

β-thalassemia is an inherited disease caused by a mutation in the gene responsible for forming the β chain of hemoglobin. The underlying pathophysiological mechanism of β-thalassemia is an imbalance in the production of α-globin and β-globin chains leading to a relative excess of α-globin. Consequently, the surplus α-globin chains produce insoluble aggregates that tend to precipitate within the developing erythroid cells. These aggregates stimulate apoptosis in the erythroid precursors, resulting in ineffective erythropoiesis and hemolytic anemia [1, 2, 3]. The clinical manifestations of β-thalassemia vary widely and are related to the difference in the combination of β alleles (a deficient [β^+^] or absent [β^0^] β-globin subunit in the hemoglobin molecule) [4].

Quantitative and functional immunological abnormalities that alter various components of the immune response are well documented in β-thalassemia major (homozygous β-thalassemia), characterized as transfusion-dependent thalassemia. Moreover, changes in cytokine profiles of innate immunity and an increase in total leukocytes and neutrophil count, which contribute to increased susceptibility to infections in these patients, have been extensively documented [2, 3, 4, 5, 6, 7]. Individuals with homozygous β-thalassemia who present milder anemia, typically not necessitating regular transfusions, and those with varying degrees of anemia requiring intermittent transfusions, are classified as β-thalassemia intermedia. These patients exhibit a spectrum of clinical manifestations ranging from severe symptoms necessitating treatment, like in thalassemia major, to mild or asymptomatic conditions, resembling thalassemia minor, according to the presence or absence of modifying genes [4].

Individuals who have inherited a single β-thalassemia allele, whether β° or β^+^, are considered to have the heterozygous thalassemia, also known as β-thalassemia trait (hBTh), and are non-transfusion-dependent. They typically exhibit low hemolysis ratios and frequently are clinically asymptomatic [3,7]. However, some hBTh individuals may display mild anemia, characterized by hypochromic and microcytic red blood cells, elevated levels of hemoglobin (Hb) A_2_, and variable increases in Hb F. In some cases, hBTh may manifest a variety of symptoms including headache, lethargy, fatigue, dizziness, and exercise intolerance, despite having hemoglobin levels within the normal range [4,8].

Despite a relatively benign presentation or even an absence of symptoms, the hBTh profile regarding inflammatory biomarkers is not well defined. Therefore, it is crucial to analyze inflammatory biomarkers, such as hematological ratios obtained from blood counts [9, 10, 11, 12].

Given the potential contribution of these indices to a more nuanced understanding of hBTh, ultimately leading to more effective patient care, this study aimed to analyze the hematological ratios obtained from blood counts of individuals with hBTh compared to a healthy control group.

## Material and methods

This retrospective observational study was approved by the Research Ethics Committees of the School of Pharmaceutical Sciences of the University of São Paulo (protocol no. 69/2012), the Federal University of São Paulo (protocol no. 69574), and Irmandade Santa Casa de Misericórdia de São Paulo Hospital (protocol no. 230.882).

Fifty patients diagnosed with heterozygous β-thalassemia (hBTh) aged from 19–84 years of both sexes were recruited from various sources, including the Hemoglobinopathies Laboratory of the Clinical Pathology Division of Clinics Hospital of State University of Campinas, the Anemia Ambulatory of the Federal University of São Paulo (UNIFESP), the Irmandade Santa Casa de Misericórdia de São Paulo Hospital, and the Hematological Ambulatory of the Faculty of Medicine and Health Sciences at the Pontifical Catholic University of São Paulo. The initial diagnosis of thalassemia was made using a complete blood count and hemoglobin electrophoresis and later confirmed by mutation type evaluations, as previously described.^13^ Individuals with β-thalassemia intermedia were excluded from this study, with only those presenting β-thalassemia minor being included. The Control Group comprised 100 healthy individuals aged from 20–82 years from the Faculty of Pharmaceutical Sciences at the State University of São Paulo, and UNIFESP, and volunteers from the city of São Paulo recruited through convenience sampling. Individuals with chronic alcoholism, active infections, pregnancy, chronic diseases, or immunosuppressive medication use, or those who had donated blood in the six months prior to the study, were excluded. Control Group individuals who consumed multivitamins, folic acid, vitamin B12, or iron were also excluded.

The data set included blood count (including natural killer [NK] cells and reticulocytes), high-sensitive C-reactive protein (CRP), interleukin (IL)-6 and IL-10, lactate dehydrogenase (LDH) activity, body mass index (BMI), age, sex, smoking, and folic acid use as previously described.^13^ Briefly, venous blood samples were obtained from each participant after an overnight fast (8–10 h). Complete blood and reticulocyte counts were determined in ethylenediaminetetraacetic acid (EDTA) whole-blood samples using a Pentra 120 Hematology Analyzer (Horiba). High-sensitivity CRP was determined by an immunoturbidimetric assay using the Roche-CRPL kit on the Cobas 8000 analyzer (Roche Diagnostics). LDH activity was determined by an enzymatic assay using the Vitros 250 analyzer (Ortho Clinical Diagnostics). IL-6 and IL-10 were determined by a multiplex immunoassay, the high-sensitivity panel T Cell Magnetic Bead Milliplex Map (EMD Millipore Corporation) on the Bio-PLex 200 analyzer (Bio-Rad Laboratories, Inc.), following the manufacturers’ protocols. The following hematological ratios were calculated from blood count data: neutrophil/lymphocyte (NLR), derived neutrophil/lymphocyte (d-NLR), lymphocyte/monocyte (LMR), platelet/lymphocyte (PLR) and neutrophil/platelet (NPR) ratios, and SII (multiplication of platelets by total neutrophils, divided by total lymphocytes), SIRI (multiplication of neutrophils by monocytes/lymphocytes) indexes, and the NNKR.

Analyses were conducted using the Statistical Package for the Social Sciences version 22.0 and GraphPad Prism software. Variables are expressed as medians and interquartile ranges, and the Mann-Whitney test was employed to compare the groups. A significance level of 5% (p-value <0.05) was used.

## Results

The general data for the groups analyzed are presented in Table 1. The BMIs of hBTh patients were similar to those of the Control Group, as well as the percentages of smokers and females, and the age.

**Table 1:** General data of the study participants.

**Variable**	**Control (*n*=100)**	**hBTh (*n*=50)**	***p*-value**
Age (y)	45.5 (32.2–58.0)	51.0 (37.0–59.7)	0.228
BMI (kg/m²)	25.5 (23.5–28.4)	25.5 (24.0 –29.4)	0.440
Female	67 (67.0)	35 (70.0)	0.710^a^
Smoker	14 (14.0)	5 (10.0)	0.487^a^
Folic acid supplementation	0	13 (26.0)	<0.001^b^
Anemia	0	38 (76.0)	<0.001^b^

hBTh, heterozygous β-thalassemia; BMI, body mass index.

a Pearson’s Chi-square.

b Likelihood ratio. Continuous variables are presented as medians with interquartile ranges. Categorical variables are expressed as the number of subjects and corresponding percentage (in parentheses). The data were subjected to the Mann-Whitney test for comparison.

The hBTh Group exhibited higher leukocyte, neutrophil, and reticulocyte counts compared to the Control Group. Regarding the interleukins evaluated, IL-6 showed higher levels in the hBTh Group than in the Control Group. The LDH activity was comparable between the hBTh patients. The detailed results of these analyses are presented in Table 2.

**Table 2:** Hematological ratios and other laboratory data of the study participants.

**Variable**	**Control (*n*=100)**	**hBTh (*n*=50)**	***p*-value**
NLR	1.38 (1.19–1.83)	1.70 (1.33–2.18)	0.010
d-NLR	1.04 (0.92–1.33)	1.33 (1.07–1.56)	0.001
LMR	4.27 (3.00–7.82)	4.14 (2.80–7.04)	0.614
PLR	0.09 (0.07–0.12)	0.09 (0.08–0.10)	0.616
NPR	16.22 (12.74–19.42)	19.87 (14.72–25.18)	0.001
SII	318 (242–395)	361 (288–459)	0.038
SIRI	691 (420–1189)	928 (507–1618)	0.056
NNKR	7.24 (4.89–8.94)	7.62 (4.30–9.01)	0.909
Leukocytes (/mm³)	6800 (5625–7600)	7200 (6250–8825)	0.011
Neutrophils (/mm³)	3456 (2866–4091)	3977 (3334–5303)	<0.001
Lymphocytes (/mm³)	2408 (1863–2921)	2448 (2086–2811)	0.652
Monocytes (/mm³)	489 (358–718)	545 (379–797)	0.304
Eosinophils (/mm³)	130 (70–216)	99 (69–166)	0.307
Basophils (/mm³)	0 (0–54)	0 (0–0)	0.044
Platelets (x 10³/mm³)	220 (187–246)	211 (179–278)	0.674
Hemoglobin (g/dL)	14.0 (13.3–15.0)	11.6 (10.6–12.4)	<0.001
Reticulocytes (%)	0.85 (0.70–1.17)	1.10 (0.85–1.75)	0.001
LDH (U/L)	426 (391–497)	428 (382–492)	0.851
CRP (mg/dL)	0.18 (0.07–0.40)	0.24 (0.10–0.45)	0.323
IL-6 (pg/mL)	0.94 (0.53–1.38)	1.19 (0.84–1.76)	0.016
IL-10 (pg/mL)	2.41 (1.07–4.46)	2.67 (1.04–4.58)	0.778

hBTh, heterozygous β-thalassemia; NLR, neutrophil/lymphocyte ratio; d-NLR, derived neutrophil/lymphocyte ratio; LMR, lymphocyte/monocyte ratio; PLR, platelet/lymphocyte ratio; NPR, neutrophil/platelet ratio; SII, systemic immuno-inflammation index; SIRI, systemic inflammation response index; NNKR, neutrophil/natural killer cells ratio; LDH, lactate dehydrogenase; CRP, C-reactive protein; IL, interleukin.

Variables are presented as median and interquartile range, and the groups were subjected to the Mann-Whitney test for comparison.

**Figure 1:**
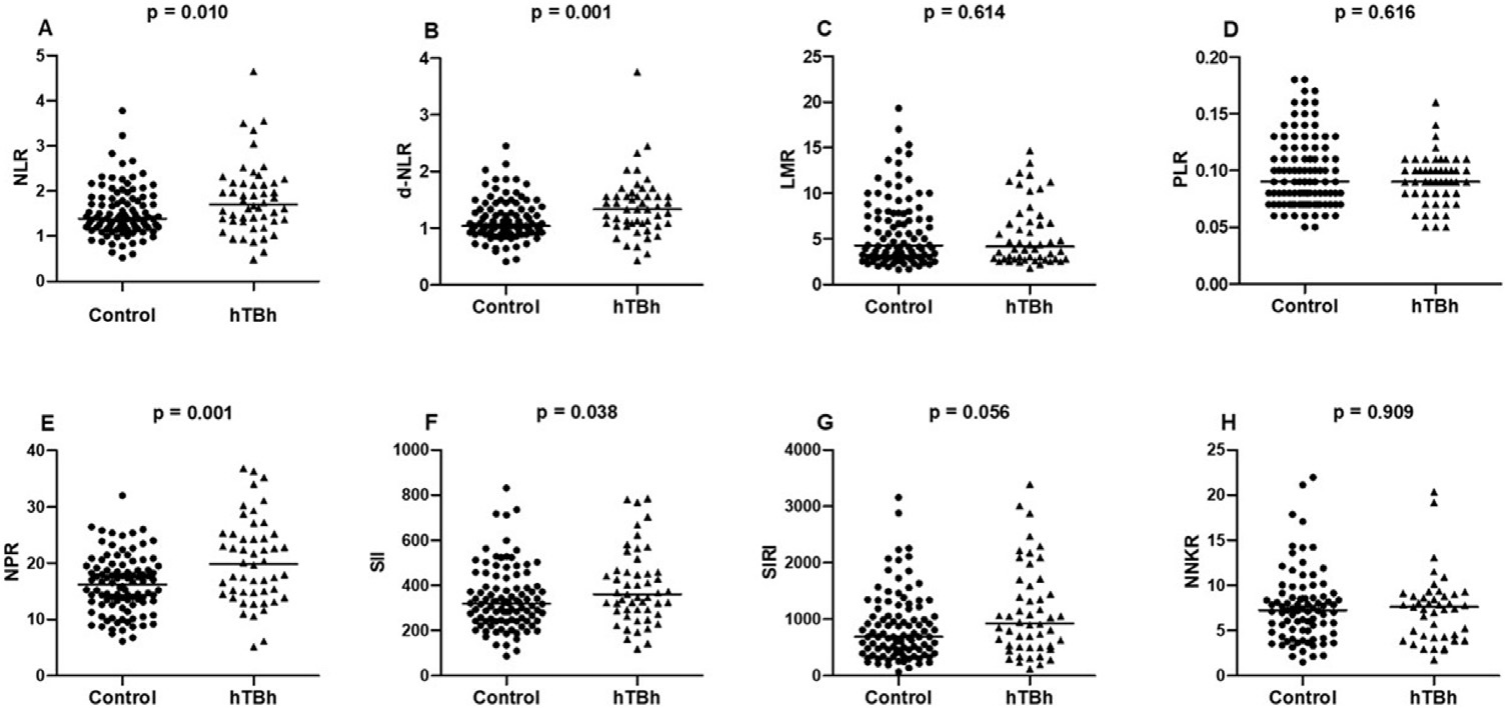
(A) neutrophil/lymphocyte ratio (NLR); (B) derived neutrophil/lymphocyte ratio (d-NLR); (C) lymphocyte/monocyte ratio (LMR); (D) platelet/lymphocyte ratio (PLR); (E) neutrophil/platelet ratio (NPR); and (F) systemic immuno-inflammation index (SII), (G) systematic inflammatory response index (SIRI); (H) neutrophil/natural killer cell ratio (NNKR) in heterozygous β-thalassemia (hBTh) subjects and the Control Group. The groups were subjected to the Mann-Whitney test for comparisons.

Furthermore, data distribution of each hematological ratio in hBTh patients and controls are shown in Figure 1.

## Discussion

To our knowledge, this is the first study to compare hematological ratios in a group of apparently healthy subjects with carriers of usually asymptomatic disorders of hematopoiesis (e.g., hBTh). Despite sometimes being considered healthy, this study demonstrates that hBTh subjects have a greater inflammatory profile compared to the Control Group.

Hematologic ratios such as NLR, d-NLR, LMR, PLR, and SII are inflammatory markers previously described as diagnostic aids for other diseases.^14^, ^15^, ^16^, ^17^, ^18^ Recent data suggest that NLR is associated with various inflammatory conditions, including diabetes mellitus, irritable bowel disease, and thyroiditis, both with subtle and overt inflammation.^18^, ^19^, ^20^, ^21^ Similarly, PLR is associated with inflammatory conditions such as liver fibrosis and cancer.^22^^,^^23^ SII, an inflammatory marker, has been used as a prognostic indicator in the follow-up of sepsis and cancer patients.^24^^,^^25^ Furthermore, SII has been studied as an aid in the diagnosis and prognosis of other diseases, including COVID-19.^18^^,^^26^, ^27^, ^28^ Most of these hematologic ratios, including SIRI, were associated with length of hospital stay and independent predictors of in-hospital mortality of patients undergoing on-pump cardiac surgery.^29^

Regarding NLR, d-NLR, and SII, there were notable differences between the hBTh and Control Group. In individuals with hBTh, there was a significant increase in the total leukocyte and neutrophil counts compared to the control subjects. Conversely, no statistically significant differences were observed in the total number of lymphocytes and platelets between the groups. Therefore, the elevated neutrophil count observed can be attributed to an inflammatory process with a subsequent increase in NLR, d-NLR, and SII ratios. These ratios may serve as potential inflammatory markers for individuals with hBTh, especially when considering neutrophil values alone.

The NLR, d-NLR, and SIRI ratios showed significant differences on comparing the Control Group and patients with hBTh, as this condition results in a chronic inflammatory process and a mild form of anemia.^7^^,^^9^ The NPR in the hBTh group was higher than in the Control Group, confirming an inflammatory state. This observation was made because the neutrophil count was increased in the hBTh Group, while the platelet count did not show any significant variation between the groups. Thus, the NPR ratio can be used as an inflammatory marker for hBTh conditions with the NPR being more indicative than its isolated parameters. In contrast, the LMR did not demonstrate any significant difference between the groups, suggesting that it may not be a reliable biomarker for monitoring inflammatory processes.

Analyses of the reticulocyte counts showed significant differences between both groups, with the findings of this study confirming that the disease involves clear hemolysis mechanisms. In hBTh, the hemolysis is less pronounced, as many patients do not present anemia, indicating hemolysis with erythropoietic compensation. One study demonstrated that the moderate reticulocytosis resulting from a mild erythrocyte response is typically sufficient to compensate for the hemolysis observed in this condition.^30^ These considerations support the view that in hBTh, the hemolysis mechanism is less severe and the erythropoietic response is more effective.

These findings are corroborated by the LDH activity. Under hemolytic conditions, evidence has shown that the quantity of this enzyme increases in the plasma.^31^ The LDH activity in both the Control and hBTh Groups was found to be comparable, a result that is consistent with the literature since hBTh is characterized by mild hemolysis.^8^

Other inflammatory markers, such as IL-6 and IL-10 and C-reactive protein, were also analyzed. The hBTh Group exhibited elevated IL-6 activity compared to the Control Group. This phenomenon can be explained by the action of macrophages that phagocytize defective red blood cells, resulting in their production of IL-6.^32^ Therefore, this result suggests that IL-6 plays a relevant role in the inflammatory response observed in this disease.

Conversely, IL-10, an anti-inflammatory cytokine that limits the immune response to pathogens,^33^ and C-reactive protein, a non-specific marker of systemic inflammatory processes,^34^ showed no significant differences between the groups analyzed. Therefore, C-reactive protein lacks prognostic value for hBTh and cannot be used as an inflammatory marker in these conditions.

It is known that in the most severe forms of β-thalassemia (major and intermedia), excess alpha chains aggregate, forming inclusions that damage cell and organelle membranes. These aggregates also induce reactive oxygen species formation, further damaging membrane proteins and lipids. Hemichromes, one of the most toxic products of unpaired α chains, binds to membranes and promotes band 3 clustering, a key membrane constituent, leading to cellular apoptosis.^4^ In patients with hBTh, hemolysis, though less intense, is still present as indicated by increased reticulocytes. This suggests that these patients undergo a similar inflammatory process to those with more severe forms of the disease, but to a lesser extent. The milder inflammatory response is likely associated to iron overload, ineffective erythropoiesis, and oxidative stress, collectively contributing to a pro-inflammatory state.

The major limitation of this study is the relatively small sample size. In cases of hBTh, most individuals are asymptomatic and do not seek medical attention. Another important limitation is the lack of clinical follow-up for hBTh individuals. However, we demonstrated that hematological ratios in this form of hemolytic anemia are more informative markers of inflammation than their isolated parameters and are more useful than traditional markers. These ratios could contribute to the management of hBTh, being easily applicable, widely available, and cost-effective.

## Conclusion

In hBTh, hematological ratios such as NLR, d-NLR and NPR and SII index demonstrated higher values than those in the Control Group, indicating a more inflammatory profile. These ratios (NLR, d-NLR, NPR, and SII) showed significant potential when applied to the hBTh Group, exhibiting a good capacity to serve as inflammatory markers when compared to isolated parameters from hemogram. Furthermore, these hematological ratios may prove valuable in managing and understanding hBTh manifestations, offering a convenient, accessible, and cost-effective alternative, since this information is calculated from blood count data, without additional analysis costs.

Further studies are required to substantiate this hypothesis, enabling the routine application of hematological indices to support medical decision-making regarding the optimal treatment strategy for individuals with hBTh, considering their chronic inflammatory state. Moreover, hematological ratios could serve as an additional tool for the preliminary assessment of individuals with suspected hBTh, a condition to be confirmed later.

## Funding

This study was financially supported by Fundação de Amparo à Pesquisa do Estado de São Paulo (FAPESP 2012/12,912–1) and Conselho Nacional de Desenvolvimento Científico e Tecnológico (CNPq 4826,412,012–6 and 401,586/2014 6), and in part by the Coordenação de Aperfeiçoamento de Pessoal de Nível Superior - Brazil (CAPES) - Finance Code 001, Brazil.

## Author Contribution

ACMC, LEO, and ALR contributed to the study design, data organization, analysis, and manuscript drafting. JAMC performed data analysis and critically revised the manuscript. MRL and GWG were responsible for sample collection, laboratory assessments, data analysis, and manuscript writing. MSF, MNNS, VLNBD, and RDC facilitated patient inclusion, collected clinical data, and reviewed the manuscript. EMGS contributed to the study design, data analysis, statistical evaluation, manuscript writing, and expert review. CP participated in study design, sample and data collection, laboratory determinations, data analysis, manuscript writing, and final review.

## Conflicts of interest

The authors declare no conflicts of interest related to this study.

## References

[1] Olivieri NF. (1999). The β-thalassemias. N Engl J Med.

[2] Origa R. (2017). β-thalassemia. Genet Med.

[3] Gluba-Brzózka A., Franczyk B., Rysz-Górzynska M., Rokicki R., Koziarska-Rosciszewska M., Rysz J. (2021). Pathomechanisms of immunological disturbances in β-thalassemia. Int J Mol Sci.

[4] Nienhuis A.W., Nathan DG. (2012). Pathophysiology and clinical manifestations of the β-thalassemias. Cold Spring Harb Perspect Med.

[5] Farmakis D., Porter J., Taher A., Domenica Cappellini M., Angastiniotis M., Eleftheriou A. (2022). 2021 Thalassaemia International Federation guidelines for the management of transfusion-dependent thalassemia. HemaSphere.

[6] Nithichanon A., Tussakhon I., Samer W., Kewcharoenwong C., Ato M., Bancroft G.J. (2020). Immune responses in beta-thalassemia: heme oxygenase 1 reduces cytokine production and bactericidal activity of human leucocytes. Sci Rep.

[7] Elsayh K.I., Mohammed W.S., Zahran A.M., Saad K. (2016). Leukocytes apoptosis and adipocytokines in children with beta thalassemia major. Clin Exp Med.

[8] Cao A., Galanello R. (2010). Beta-thalassemia. Genet Med.

[9] Aly M.M., Meshref T.S., Abdelhameid M.A., Ahmed S.A., Shaltout A.S., Abdel-Meniem A.E. (2021). Can hematological ratios predict outcome of covid-19 patients? A multicentric study. J Blood Med.

[10] Fois A.G., Paliogiannis P., Scano V., Cau S., Babudieri S., Perra R. (2020). The systemic inflammation index on admission predicts in-hospital mortality in COVID-19 patients. Molecules.

[11] Jiang S., Wang S., Wang Q., Deng C., Feng Y., Ma F. (2021). Systemic inflammation response index (SIRI) independently predicts survival in advanced lung adenocarcinoma patients treated with first-generation EGFR-TKIs. Cancer Manag Res.

[12] Zhou Q., Su S., You W., Wang T., Ren T., Zhu L. (2021). Systemic inflammation response index as a prognostic marker in cancer patients: A systematic review and meta-analysis of 38 cohorts. Dose Response.

[13] Paniz C, Lucena MR, Bertinato JF, Santos MNN, Gomes GW, Figueiredo MS, et al. Serum folate and cytokines in heterozygous β-thalassemia. Int J Lab Hematol. 42(6):718-726. 10.1111/ijlh.13287 32662566

[14] Rinaldi I., Hamonangan R., Azizi M.S., Cahyanur R., Wirawan F., Fatya A.I. (2021). Diagnostic value of neutrophil lymphocyte ratio and D-Dimer as biological markers of deep vein thrombosis in patients presenting with unilateral limb edema. J Blood Med.

[15] Köse N., Yıldırım T., Akın F., Yildirim S.E., Altun I. (2020). Prognostic role of NLR, PLR, and LMR in patients with pulmonary embolism. Bosn J Basic Med Sci.

[16] Ying H.Q., Deng Q.W., He B.S., Pan Y.Q., Wang F., Sun H.L. (2014). The prognostic value of preoperative NLR, d-NLR, PLR and LMR for predicting clinical outcome in surgical colorectal cancer patients. Med Oncol.

[17] Soliman W.M., Sherif N.M., Ghanima I.M., El-Badawy MA. (2018). Neutrophil to lymphocyte and platelet to lymphocyte ratios in systemic lupus erythematosus: relation with disease activity and lupus nephritis. Reumatol Clin.

[18] Fernandes N.F., Costa I.F., Pereira K.N., de Carvalho J.A.M., Paniz C. (2023). Hematological ratios in coronavirus disease 2019 patients with and without invasive mechanical ventilation. J Investig Med.

[19] Bilgin S., Aktas G., Kocak M.Z., Atak B.M., Kurtkulagi O., Duman T.T. (2020). Association between novel inflammatory markers derived from hemogram indices and metabolic parameters in type 2 diabetic men. Aging Male.

[20] Aktas G., Duman T.T., Atak B.M., Kurtkulagi O., Bilgin S., Basaran E. (2020). Irritable bowel syndrome is associated with novel inflammatory markers derived from hemogram parameters. Fam Med Prim Care Rev.

[21] Aktas G., Sit M., Dikbas O., Erkol H., Altinordu R., Erkus E. (2017). Elevated neutrophil-tolymphocyte ratio in the diagnosis of Hashimoto’s thyroiditis. Rev Assoc Med Bras.

[22] Kosekli MA. (2022). Mean platelet volume and platelet to lymphocyte count ratio are associated with hepatitis B-related liver fibrosis. Eur J Gastroenterol Hepatol.

[23] Atak B.M., Kahveci G., Bilgin S., Kurtkulagi O., Kosekli MA. (2021). Platelet to lymphocyte ratio in differentiation of benign and malignant thyroid nodules. Exp Biomed Res.

[24] Zhang Y., Sun Y., Zhang Q. (2020). Prognostic value of the systemic immune-inflammation index in patients with breast cancer: a meta-analysis. Cancel Cell Int.

[25] Pedersen S.F., Ho Y. (2020). SARS-CoV-2: a storm is raging. J Clin Invest.

[26] Fois A.G., Paliogiannis P., Scano V., Cau S., Babudieri S., Perra R. (2020). The systemic inflammation index on admission predicts in-hospital mortality in COVID-19 patients. Molecules.

[27] Huang Y., Chen Y., Zhu Y., Wu Q., Yao C., Xia H. (2021). Postoperative systemic immune-inflammation index (SII): a superior prognostic factor of endometrial cancer. Front Surg.

[28] Liu X., Guan G., Cui X., Liu Y., Liu Y., Luo F. (2021). Systemic immuneinflammation index (SII) can be an early indicator for predicting the severity of acute pancreatitis: a retrospective study. Int J Gen Med.

[29] Rödel A.P.P., Fernandes Y.M., Brisolara J.V., de Carvalho J.A.M., Moresco RN. (2025). Role of Preoperative Inflammatory Blood Cell Indexes as a Postoperative Risk Predictor Among Patients Undergoing On-Pump Cardiac Surgery. Int J Lab Hematol.

[30] Carvalho LB. (2003).

[31] Henry JB. (2008).

[32] Pfefferlé M., Ingoglia G., Schaer C.A., Yalamnoglu A., Buzzi R., Dubach I.L. (2020). Hemolysis transforms liver macrophages into anti-inflammatory erythrophagocytes. J Clin Invest.

[33] Saraiva M., Vieira P., O'Garra A. (2020). Biology and therapeutic potential of interleukin-10. J Exp Med.

[34] Villacorta H., Masetto A.C., Mesquita ET. (2007). Proteína C-reativa: marcador inflamatório com valor prognóstico em pacientes com insuficiência cardíaca descompensada. Arq Bras Cardiol.

